# Prediction of enzyme function by combining sequence similarity and protein interactions

**DOI:** 10.1186/1471-2105-9-249

**Published:** 2008-05-27

**Authors:** Jordi Espadaler, Narayanan Eswar, Enrique Querol, Francesc X Avilés, Andrej Sali, Marc A Marti-Renom, Baldomero Oliva

**Affiliations:** 1 Laboratori de Bioinformàtica Estructural (GRIB), Departament de Ciències Experimentals i de la Salut, Universitat Pompeu Fabra-IMIM, 08003-Barcelona, Catalonia, Spain; 2 Institut de Biotecnologia i Biomedicina and Departament de Bioquímica, Universitat Autònoma de Barcelona, 08193-Bellaterra (Barcelona), Spain; 3 Departments of Biopharmaceutical Sciences and Pharmaceutical Chemistry, University of California, San Francisco, CA 94158-2330, USA; 4 California Institute for Quantitative Biomedical Research, University of California, San Francisco, CA 94158-2330, USA; 5 Structural Genomics Unit, Bioinformatics Department, Centro de Investigación Príncipe Felipe (CIPF), 46012-Valencia, Spain

## Abstract

**Background:**

A number of studies have used protein interaction data alone for protein function prediction. Here, we introduce a computational approach for annotation of enzymes, based on the observation that similar protein sequences are more likely to perform the same function if they share similar interacting partners.

**Results:**

The method has been tested against the PSI-BLAST program using a set of 3,890 protein sequences from which interaction data was available. For protein sequences that align with at least 40% sequence identity to a known enzyme, the specificity of our method in predicting the first three EC digits increased from 80% to 90% at 80% coverage when compared to PSI-BLAST.

**Conclusion:**

Our method can also be used in proteins for which homologous sequences with known interacting partners can be detected. Thus, our method could increase 10% the specificity of genome-wide enzyme predictions based on sequence matching by PSI-BLAST alone.

## Background

While the amount of genome sequence information is increasing exponentially, the annotation of protein sequences remains a problem, both in terms of quality and quantity [1]. Bioinformatics-based annotation of uncharacterized proteins is still one of the most challenging problems in biology [2]. The classical approach involves transfer of annotation from a functionally characterized protein to its functionally uncharacterized homologs. Although, several studies have highlighted the limitations of such methods[1,3,4], they have been extensively used on annotating proteins and in particular enzymes [5,6].

About half of all proteins with experimentally characterized functions have enzymatic activity, making enzymes the largest single class of proteins [5]. The Enzyme Commission (EC) uses four numbers (integers) separated by periods to classify the functions of enzymes [7]. The first three digits describe the overall type of an enzymatic reaction, while the last digit represents the substrate specificity of the catalyzed reaction. The accuracy of transferring an enzymatic annotation between two globally aligned protein sequences has been reported to significantly drop under 60% sequence identity [6]. To address this limitation, we introduce for first time an approach that combines sequence similarity search and comparative protein interaction data to increase the confidence in automatic enzyme annotation. Our hypothesis relies in the rationale that homologous proteins perform similar functions when associated with similar interacting partners. Therefore, two sufficiently similar proteins with common interactions should probably share the same first three EC numbers (common enzymatic function).

Protein-protein interactions have been used for functional annotation by several different approaches, such as Markov random fields [8,9], minimization of interactions among proteins from different functional categories [10], message passing algorithms [11], neighbourhood weights [12], network-flow algorithms [13], the number of common interaction partners [14], and the combination of common interaction partners and common domains [15]. However, the results from some of the existing methods are limited by the need to know the function of interacting partners to annotate the query protein. This limitation is even more dramatic for annotating enzymatic function because enzymes usually do not interact with other enzymes of the same function. Possible exceptions are enzymes involved in proteolytic (*e.g*., clotting cascade) or signalling cascades (*e.g*., MAP-kinase cascades). Moreover, the benchmarks of such methods did not account for the fact that protein families have different distributions in different genomes. Therefore, the accuracy obtained for a method in a given genome can be biased due to the specific representation of protein families within the genome. This problem has been already addressed by averaging the results for each protein families according to PFAM [5,6] or by describing the degree of function conservation *versus *sequence identity [15,16].

Next, we outline the impact of protein interactions on annotation based on sequence similarity alone (Results). Then, we discuss the implications of our combined approach for functional annotation in general (Discussion). Finally, we describe our approach in detail (Methods).

## Results

### Approach overview

As mentioned above, our method relies in the rationale that homologous proteins perform similar functions when associated with similar interacting partners. Moreover, based on the observation that homologous proteins interact with similar partners in similar ways [17], our method applies an additional transference of interactions by means of homology (we name this as expansion of interactions [18]). Thus, if two proteins do not share exactly the same interaction, but interact with homologous proteins, our method is still applicable. Our method, called ModFun, has been trained and tested using a benchmark set of proteins with known enzymatic function and known interactions or with detectable homologous with known interactions. The benchmark set was randomly split in two sets with similar number of proteins. The first set (for training) was used to find the best criteria of similarity between sequences for the transference of interactions (expansion) and the threshold to filter the predictions using common interactions. The second set (for testing) was used to test the increase in accuracy for function assignment of our approach with respect to PSI-BLAST.

### Analysis of the data set

**Figure 1:**
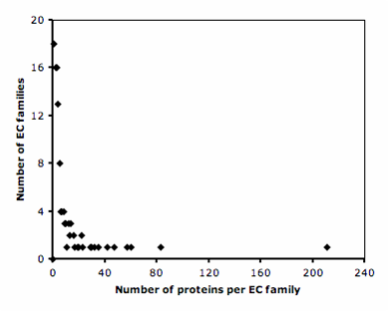
Frequency distribution of EC families within the SP-EC dataset, as defined by the first three EC digits.

The set SP-DIP (Methods) contains 1,227 enzymes and 2,663 non-enzymes. According to the PFAM domain architecture [19], enzymes and non-enzymes were grouped into 630 and 1,296 homologous families, respectively. The enzyme PFAM families cluster in 116 EC families according to the first three digits of the EC code. Approximately, half of these EC families have between 1 and 4 sequences (Figure 1). However, the six most populated EC families have more than 40 representatives each, accounting for 40% of all enzymes in the test set. These EC families are: (i) kinases using alcohol groups as acceptors, EC 2.7.1; (ii) nucleotidyl transferases, EC 2.7.7; (iii) phosphatases, EC 3.1.3; (iv) ATPases catalyzing transmembrane movement of substances, EC 3.6.3; (v) amino-acid ligases, EC 6.1.1; and (vi) dehydrogenases acting on CH-OH groups using NAD/NADP as acceptor, EC 1.1.1. Therefore, to perform an unbiased statistical analysis, the degree of conservation was averaged over each family.

### Parameters optimization

A pair of homologous proteins P1 – P2 can be related through common interaction partners. The method requires that the proteins used in the expansion procedure (P1 and P1') perform the same enzymatic function. Previous work suggested 60% identity of a BLAST alignment as a reliable cutoff for the conservation of the enzymatic function as defined by the first three EC digits [6]. However, to a lesser extent the BLAST e-value [20] has also been reported as indicative of enzymatic function conservation [6]. Therefore, to expand an interaction between two proteins to their homologs, both sequence identity and BLAST e-value cutoffs need to be fulfilled, hereby referred as the expansion cutoff. We have explored expansion cutoffs ranging from 20% to 70% for the sequence identity and 0.0001 for the e-value.

Protein interactions were used in combination with homology detection only for sequence pairs below a certain sequence identity cutoff, referred as the filter cutoff. We have explored filter cutoffs ranging from 25% to 65% sequence identity.

**Figure 2:**
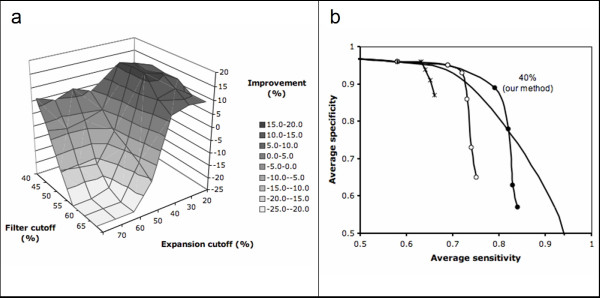
**a **Relative improvement over PSI-BLAST as a function of the parameters used (Methods). **b **ROC curves obtained for PSI-BLAST and ModFun, using different expansion and filter cutoffs (solid line, PSI-BLAST; filled circles, ModFun with expansion cutoff of 40% and filter cutoff of 55%; empty circles, ModFun with expansion cutoff of 50% and filter cutoff of 55%; stars, ModFun without expansion nor filter). Labels in the plot indicate specificity and sensitivity at the 40% identity threshold for PSI-BLAST and ModFun with optimal parameters (expansion cutoff of 40%, filter cutoff of 55%).

The data set was randomly split into training and testing sets of equal size. Using the training set, ROC curves were obtained for PSI-BLAST and for our method (ModFun) using different expansion and filter cutoff values. For each combination of expansion and filter cutoff values, the minimum distance between the ROC curve and the upper right corner of the plot (maximum specificity and sensitivity) was measured. The relative improvement with respect to PSI-BLAST was maximized to define the best results (Figure 2a). The corresponding optimal cutoff values were 40% sequence identity and 0.0001 e-value for the expansion cutoff and 55% sequence identity for the filter cutoff.

### ROC Analysis and Validation

At 40% sequence identity threshold, measured from the PSI-BLAST alignment, ModFun was able to annotate enzymes from the training set with 10% higher specificity than PSI-BLAST for the sensitivity of 80% (Figure 2b). The utility of the expansion procedure is further demonstrated by comparing the ROC curves obtained for different expansion cutoffs. For instance, for a specificity of 90%, the expansion procedure increases the coverage from 63% (no expansion) to 80% (optimal parameters).

**Figure 3:**
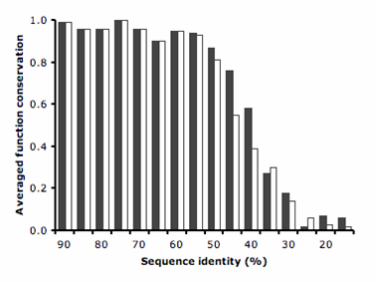
Averaged function conservation as a function of the sequence identity (empty bars, PSI-BLAST; filled bars, ModFun with optimal parameters).

The largest benefit of using ModFun for functional annotation occurs for pairs of sequences that align with sequence identities between 40 and 55% (Figure 3). At this level of sequence identity, the gain in specificity is, on average, ~27%. The differences between ModFun and PSI-BLAST are small for pairs of sequences that align with less than 35% or more than 60% sequence identity. However, about one third of related pairs of enzymes from *S. cerevisiae *have sequence identity within the 40–55% range. Similar results were obtained without averaging by family (see Additional file 1). The improvement of ModFun with respect to PSI-BLAST with and without averaging by family could not be statistically distinguished using a Wilcoxon signed-rank test.

**Figure 4:**
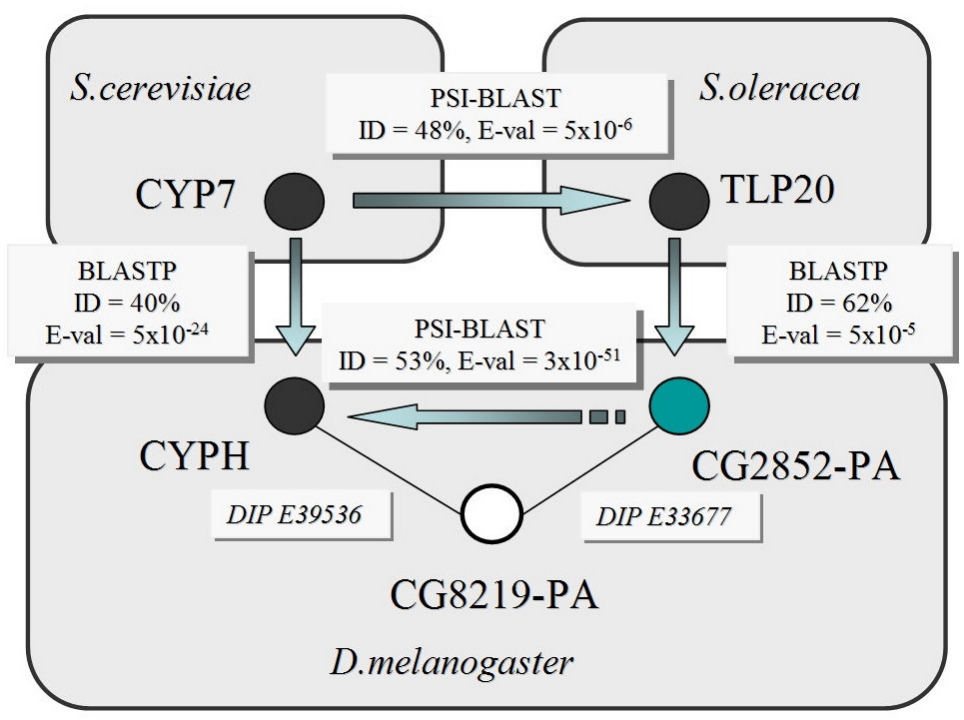
**Proteins with the same enzymatic function from two different organisms can be related by means of the known interactions of their homologs in a third organism.** Lines represent reported protein interactions; arrows represent sequence similarity; filled circles represent proteins with known enzymatic function (EC 5.2.1.8); empty circles represent proteins with no annotation.

The ability of ModFun to successfully identify protein pairs with the same enzymatic function, even using protein interaction data from other organisms than the query, is illustrated by the example of a peptidyl-prolyl cis-trans isomerase (EC 5.2.1.8) from yeast (CYP7). A PSI-BLAST search using the SP-EC database identifies TLP20, a peptidyl-prolyl cis-trans isomerase from *Spinacia oleracea*, as a putative homolog of CYP7. A BLAST search using the DIP-SP database finds putative homologs for both proteins in *Drosophila melanogaster *(with sequence identity higher than 40% and an e-value smaller than 0.0001). Among these homologs, two of them (CYPH and CG2852-PA) have a common interacting partner, the CG8219-PA open reading frame. Therefore, CYP7 and TLP20, which belong to different organisms, can be related by sequence similarity and protein interactions by means of homologous proteins in a third organism that have a common interacting partner (Figure 4). Moreover, we predict that the open reading frame CG2852-PA from Drosophila performs a function similar to those of CYP7, TLP20, and CYPH (the latter also being a known peptidyl-prolyl cis-trans isomerase).

### Statistical significance of the enrichment by ModFun

The ratio of correct functional assignments (using the first three EC digits) in DIP-SP is 1.5% and increases to 3.5% when using proteins sharing common interactions with the query sequence (Methods). Such result indicates that there is an enrichment of the proteins with the same enzymatic function within the set of relatives with common interacting partners. To quantify the statistical significance of such enrichment, we compared the enrichment for each query using the Wilcoxon test [21] against 100 randomly selected sets of interactions with the same number of relatives from DIP-SP. The corresponding p-value (< 7 × 10^-52^) quantifies the statistical significance of enrichment in the set of relatives by means of common interactions.

### Impact of our approach on annotating the genome of *S. cerevisiae*

**Figure 5:**
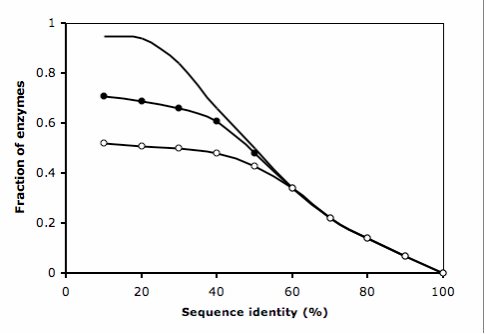
Fraction of known enzymes in the *S. cerevisiae *proteome finding a correct sequence match (as defined by the first three EC digits) as a function of the sequence identity threshold (solid line, PSI-BLAST; filled circles, ModFun; empty circles, ModFun without using interaction data from yeast).

All *S. cerevisiae *proteins with EC numbers (1,186 enzymes) were functionally annotated with PSI-BLAST and ModFun by searching against the ENZYME database. At the 40% sequence identity cutoff, the PSI-BLAST search correctly annotated 66% of the proteins in the set by transferring the correct three-digit EC number from the closest match (Figure 5). The ModFun filtering method was then applied to about one third of all the sequences in the set (284 proteins) that resulted in a PSI-BLAST hit, which alignment ranged between 40 and 55% sequence identity. ModFun found a correct match for 225 of these 284 sequences. Therefore, ~19% of the initial sequences were correctly annotated only after our filtering method was applied. ModFun can still be applied by relying only on interaction data from organisms other than the query organism. For example, after removing all interaction data for *S. cerevisiae proteins*, 71 enzymes out of the 225 still could be correctly annotated by ModFun.

## Discussion

We described, implemented, and tested a method that uses information about sequence similarity and protein-protein interactions to perform enzyme annotations between remotely related protein sequences. The method was tested on a set of proteins with known interactions containing 1,227 enzymes and 2,663 non-enzymes. Most of the existing methods have been tested by application to the *S. cerevisiae *proteome [8-10,13-15]. In this work, we have taken advantage of available protein interaction data from several organisms to test our approach. Although *S. cerevisiae *accounts for a large fraction of the 1,227 enzymes used in the SP-DIP set of known interactions (54%), other organisms were also represented (*i.e*., *E. coli*, *H. sapiens*, *D. melanogaster*, and *H. pylori *with 9%, 9%, 8%, and 7% of the sequences, respectively).

Previous studies have stressed the need to compensate for overrepresented and underrepresented protein families to obtain reliable estimates of the first three digits of an enzymatic function [5,6]. Members of an overrepresented protein family are more likely to find pairs from the same family, therefore yielding a high number of true positives. Moreover, since some protein families account for a larger fraction of the dataset than other families, statistics obtained from these families could bias the general statistics towards higher values of function conservation between pairs. In this work, we have addressed this issue by averaging our results within protein families. The results show that considering protein interactions increases the degree of enzyme function conservation for sequence pairs in the 40–55% identity range, as calculated by PSI-BLAST. Therefore, annotation transfers may be performed with increased confidence between such sequence pairs if similar interacting partners are found. For pairs with higher percentage of identity (>50%), sequence similarity alone is a good indication of function conservation (*i.e*., conservation of at least the first three EC digits).

A genome-wide test was performed for the *S. cerevisiae *sequences, for which abundant protein interactions data is available. By using ModFun, ~19% of all known enzymes in the yeast genome would have benefited from the increase in the confidence of their functional annotations. Moreover, the results show that our method can be applied to proteins without known interactions, *via *an "expansion" procedure based on known interactions of their close homologs. Because proteins involved in the expansion should perform the same enzymatic function, only homologs above 60% identity by BLAST should be considered in the expansion [6]. However, here we show that homologs with 40% identity can be used, as long as the e-value of BLAST is smaller than 0.0001. For example, without using interaction data from yeast, 6% of all known yeast enzymes still benefit from the expansion.

Although not all protein interactions need to be determined in order to achieve full coverage by our method, the increase of the number of experimentally determined protein-protein interactions will likely result in a larger applicability of ModFun. In this work, we have also shown that the identification of only one similar interacting partner for a low sequence identity pair of proteins is enough to increase the reliability of the annotation transfer. Moreover, a more complete knowledge of the sets of interacting partners is likely to improve the accuracy of ModFun by allowing the scoring of protein pairs by the number of similar interaction partners, in addition to their sequence similarity.

In conclusion, ModFun provides a higher confidence in functional annotation from sequence than sequence-based methods alone. In particular, about 20% of the enzymes would be incorrectly predicted by using only PSI-BLAST.

## Methods

### Datasets

To test our approach, we relied on the DIP database (Dec 2004 release) [22], the ENZYME database (release 36.0, Jan 2005) [7], the InterPro database (release 9.0, Feb 2005) [23], and the UniProt database (release 9.0, Feb 2005) [24].

Proteins with EC-codes were extracted from the SWISSPROT subset of UniProt. We excluded those proteins that (i) have EC numbers with undetermined digits; (ii) have more than one EC number; and (iii) are annotated as "probable", "hypothetical", "putative", "by similarity", "by homology" or "fragment" in the SWISSPROT keyword record [5,6]. These criteria resulted in the SP-EC dataset containing 49,885 protein sequences.

Additionally, we extracted proteins from the SWISSPROT database that (i) have known interactions in the DIP database, by means of their "AC" codes; (ii) have PFAM mappings in InterPro; and, (iii) are not annotated with keywords such as "probable", "hypothetical", "putative", "by similarity", "by homology" or "fragment". The resulting subset of 3,890 SWISSPROT entries (*i.e*., SP-DIP) included 2,663 proteins that do not have an EC code (*i.e*., considered non-enzymes) and 1,227 proteins that were present in the SP-EC dataset (*i.e*., considered enzymes).

### Sequence search

To test our procedure, profiles were built for each protein in the SP-DIP dataset by running the PSI-BLAST program [20] with default parameters against UniProt [24] for three iterations (or up to convergence). Enzyme-enzyme and enzyme-non-enzyme pairs were collected by searching with these profiles against the SP-DIP dataset. The outputs of the PSI-BLAST searches were filtered to remove self-matches and alignments shorter than 30 residues.

### Relating sequence pairs through interacting partners

**Figure 6:**
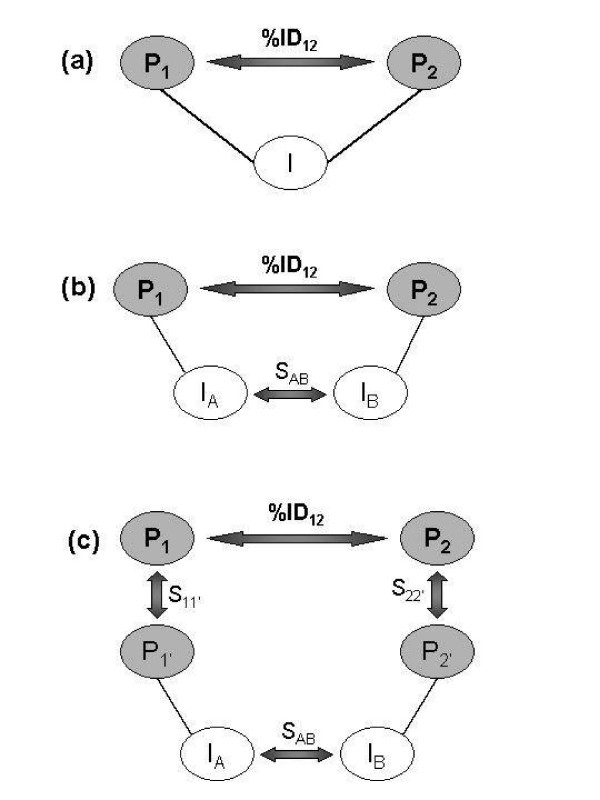
**Relating a protein pair P_1 _– P_2 _through sequence similarity and protein interactions: a) protein pair linked through a common interacting partner; b) protein pair linked through similar interacting partners; c) protein pair linked through expansion.** Lines represent reported protein interactions; arrows represent sequence similarity.

A protein interaction network can be represented by a graph with nodes as proteins and edges as protein interactions. In such a graph, a set of proteins connected to protein X (*i.e*., physically interacting with X) is named "partners of X". Figure 6 summarizes three scenarios of sequence pairs *P*_1_*-P*_2 _related through interacting partners: (a) a sequence pair related through a common interaction partner *I*; (b) a sequence pair related through similar interaction partners *I*_1 _and *I*_2_; (c) a sequence pair related through the interaction partners of their homologues *P*_1' _and *P*_2'_, hereby referred as expansion [18]. Sequence similarity was determined here by comparing each sequence in the DIP-SP to all the remaining sequences in DIP-SP by BLASTP. Two sequences were considered to be similar if aligned with an e-value ≤ 0.0001.

### Grouping of enzymes into families

Sequences from SP-DIP dataset were classified into 1,926 families according to their PFAM domain architecture. Families containing enzyme sequences were further split according to their first three EC digits. These families are referred to as EC families.

### Family-averaged sensitivity and specificity

For each query, only the sequence pair with the highest degree of sequence identity was used for this study. Averaged specificity was calculated as described by Tian and Skolnick [6]. Briefly, query-enzyme pairs above a sequence identity threshold (*e.g*., 40%) were collected for a given family *H*, and its specificity was calculated as:

$$Spec_{H,i\geq 40}=\frac{F_{H,i\geq 40}}{P_{H,i\geq 40}}$$

where *F*_*H*, *i *_is the number of pairs with the same function as defined by the first three EC digits (true positive pairs), and *P*_*H*, *i *_is the number of pairs with sequence identity above the threshold *i *(true positive plus true negative pairs). The averaged specificity was calculated as:

$$AvSpec_{i\geq 40}=\frac{\sum_{H=1}^{N}Spec_{H,i\geq 40}}{N_{i\geq 40}}$$

where *N*_*i *_is the total number of families finding sequence matches above threshold *i*, and *N *is the total number of families in the set.

Similarly, family sensitivity was calculated as:

$$Sens_{H,i\geq 40}=\frac{F_{H,i\geq 40}}{n_{H}}$$

where *n*_*H *_is the total number of sequences in family *H*. Finally, the averaged sensitivity was calculated as:

$$AvSens_{i\geq 40}=\frac{\sum_{H=1}^{N}Sens_{H,i\geq 40}}{N_{i\geq 40}}$$

### Parameter optimization

Optimal values for the expansion and filter identity cutoffs were selected on the basis of the relative improvement over PSI-BLAST. This improvement was defined as 100 × (D_1 _- D_2_)/D_2_, where D_1 _and D_2 _are the minimum distance of the ROC curve to the upper right corner of the plot for our method (ModFun) and PSI-BLAST, respectively.

### Enzyme function conservation as a function of sequence identity

For each query, only the sequence pair with the highest sequence identity was used for this study. Given a family of homologous proteins *H*, query-enzyme pairs falling in a certain sequence identity range *i *(*e.g*. 40–45%) were collected. We calculate the degree of function conservation in a similar way to specificity, but for the sequence identity interval [40%, 45%), instead of a threshold:

$$Cons_{H,45>i\geq 40}=\frac{F_{H,45>i\geq 40}}{P_{H,45>i\geq 40}}$$

Also, we calculated the average degree of conservation as:

$$AvCons_{45>i\geq 40}=\frac{\sum_{H=1}^{N}Cons_{H,45>i\geq 40}}{N_{45>i\geq 40}}$$

## Authors' contributions

JE performed the research and developed the software. AS, MAM-R and BO conceived the idea, analyzed the results and provided scientific guidance. JE, AS, MAM-R and BO wrote the manuscript. EN, FXA and EQ provided databases and scientific support. All authors read and accepted the manuscript.

## Supplementary Material

Additional file 1 Percentage of correct assignments of the first three EC digits as a function of the sequence identity (empty bars, PSI-BLAST; filled bars, ModFun with optimal parameters) Click here for file

## References

[B1] Iliopoulos I, Tsoka S, Andrade MA, Enright AJ, Carroll M, Poullet P, Promponas V, Liakopoulos T, Palaios G, Pasquier C, Hamodrakas S, Tamames J, Yagnik AT, Tramontano A, Devos D, Blaschke C, Valencia A, Brett D, Martin D, Leroy C, Rigoutsos I, Sander C, Ouzounis CA (2003). Evaluation of annotation strategies using an entire genome sequence. Bioinformatics.

[B2] Friedberg I (2006). Automated protein function prediction--the genomic challenge. Brief Bioinform.

[B3] Valencia A, Pazos F (2002). Computational methods for the prediction of protein interactions. Curr Opin Struct Biol.

[B4] Devos D, Valencia A (2000). Practical limits of function prediction. Proteins.

[B5] Rost B (2002). Enzyme function less conserved than anticipated. J Mol Biol.

[B6] Tian W, Skolnick J (2003). How well is enzyme function conserved as a function of pairwise sequence identity?. J Mol Biol.

[B7] Bairoch A (2000). The ENZYME database in 2000. Nucleic Acids Res.

[B8] Letovsky S, Kasif S (2003). Predicting protein function from protein/protein interaction data: a probabilistic approach. Bioinformatics.

[B9] Deng M, Chen T, Sun F (2004). An integrated probabilistic model for functional prediction of proteins. J Comput Biol.

[B10] Vazquez A, Flammini A, Maritan A, Vespignani A (2003). Global protein function prediction from protein-protein interaction networks. Nat Biotechnol.

[B11] Leone M, Pagnani A (2005). Predicting protein functions with message passing algorithms. Bioinformatics.

[B12] McDermott J, Bumgarner R, Samudrala R (2005). Functional annotation from predicted protein interaction networks. Bioinformatics.

[B13] Nabieva E, Jim K, Agarwal A, Chazelle B, Singh M (2005). Whole-proteome prediction of protein function via graph-theoretic analysis of interaction maps. Bioinformatics.

[B14] Samanta MP, Liang S (2003). Predicting protein functions from redundancies in large-scale protein interaction networks. Proc Natl Acad Sci U S A.

[B15] Okada K, Kanaya S, Asai K (2005). Accurate extraction of functional associations between proteins based on common interaction partners and common domains. Bioinformatics.

[B16] Espadaler J, Romero-Isart O, Jackson RM, Oliva B (2005). Prediction of protein-protein interactions using distant conservation of sequence patterns and structure relationships. Bioinformatics.

[B17] Aloy P, Stark A, Hadley C, Russell RB (2003). Predictions without templates: new folds, secondary structure, and contacts in CASP5.

[B18] Espadaler J, Aragues R, Eswar N, Marti-Renom MA, Querol E, Aviles FX, Sali A, Oliva B (2005). Detecting remotely related proteins by their interactions and sequence similarity. Proc Natl Acad Sci U S A.

[B19] Bateman A, Coin L, Durbin R, Finn RD, Hollich V, Griffiths-Jones S, Khanna A, Marshall M, Moxon S, Sonnhammer EL, Studholme DJ, Yeats C, Eddy SR (2004). The Pfam protein families database. Nucleic Acids Res.

[B20] Altschul SF, Madden TL, Schaffer AA, Zhang J, Zhang Z, Miller W, Lipman DJ (1997). Gapped BLAST and PSI-BLAST: a new generation of protein database search programs. Nucleic Acids Res.

[B21] Wilcoxon F (1945). Individual Comparisons by Ranking Methods. Biometrics.

[B22] Salwinski L, Miller CS, Smith AJ, Pettit FK, Bowie JU, Eisenberg D (2004). The Database of Interacting Proteins: 2004 update. Nucleic Acids Res.

[B23] Mulder N, Apweiler R (2007). InterPro and InterProScan: Tools for Protein Sequence Classification and Comparison. Methods Mol Biol.

[B24] Bairoch A, Apweiler R, Wu CH, Barker WC, Boeckmann B, Ferro S, Gasteiger E, Huang H, Lopez R, Magrane M, Martin MJ, Natale DA, O'Donovan C, Redaschi N, Yeh LS (2005). The Universal Protein Resource (UniProt). Nucleic Acids Res.

