# Supplementary material for: Prediction of enzyme function by combining sequence similarity and protein interactions
Source: BMC Bioinformatics. 2008 May 27;9:249. doi: 10.1186/1471-2105-9-249 (PMC2430716; doi:10.1186/1471-2105-9-249)
Supplement: Additional file 1 — Percentage of correct assignments of the first three EC digits as a function of the sequence identity (empty bars, PSI-BLAST; filled bars, ModFun with optimal parameters) [file 1471-2105-9-249-S1.doc]

***Additional File 1*** *Percentage of correct assignement of the first three EC digits as a function of the sequence identity (empty bars, PSI-BLAST; filled bars, ModFun with optimal parameters).*
